# The Study of Learning Computer Programming for Students with Medical Fields of Specification—An Analysis via Structural Equation Modeling

**DOI:** 10.3390/ijerph19106005

**Published:** 2022-05-15

**Authors:** Ching-Hsiang Lai, Yan-Kwang Chen, Ya-huei Wang, Hung-Chang Liao

**Affiliations:** 1 Department of Medical Informatics, Chung Shan Medical University, Taichung 40201, Taiwan; liay@csmu.edu.tw; 2 Information Technology Office, Chung Shan Medical University Hospital, Taichung 40201, Taiwan; 3 Department of Distribution Management, National Taichung University of Science and Technology, Taichung 40401, Taiwan; ykchen@nutc.edu.tw; 4 Department of Applied Foreign Languages, Chung Shan Medical University, Taichung 40201, Taiwan; 5 Department of Medical Education, Chung Shan Medical University Hospital, Taichung 40201, Taiwan; 6 Department of Health Policy and Management, Chung Shan Medical University, Taichung 40201, Taiwan; 7 Department of Medical Management, Chung Shan Medical University Hospital, Taichung 40201, Taiwan

**Keywords:** computer programming learning attitude (CPLA), computational thinking (CT) perspectives, programming empowerment (PE), structural equation modeling (SEM)

## Abstract

In this study, the authors constructed structural equation models in order to determine the relationship between students’ learning attitudes and their computational thinking perspectives and programming empowerment. The purpose is to understand students’ perceived competence to use computational thinking effectively, along with their computer programming learning attitude regarding the C++ programming language for one semester (2 hours per week, 36 total learning hours). A total of 495 students specializing in the medical field participated in the study. Structural equation models were constructed according to three adapted scales: the computer programming learning attitude scale, the computational thinking perspectives scale, and the programming empowerment scale. The computer programming learning attitude scale is based on three factors: willingness, negativity, and necessity. The computational thinking perspectives scale also considers three factors: the ability to express, the ability to connect, and the ability to question. The programming empowerment scale is composed of four factors: meaningfulness, impact, creative self-efficacy, and programming self-efficacy. The results showed that a positive learning attitude will positively affect computational thinking perspectives and programming empowerment. However, when students have a negativity attitude, feeling that they are being forced to learn the C++ programming language, their computational thinking perspectives and programming empowerment will be negatively affected. In order to promote students’ learning attitude, various teaching strategies, teaching curriculum design, and pedagogy design could be further explored.

## 1. Introduction

In Taiwan, in response to AI globalization, most universities have listed computer programming as a required course since, regardless of the subject, computer programming could be a necessary skill for the future [1]. Otherwise, students may have conceptual gaps when they must use computers in their field of study in the future. However, although it is necessary to learn computer programming, many students have difficulty learning it because they struggle with logic, mathematics, and problem-solving strategies [2,3]. Hence, most students are challenged by the computer programming course, which, in turn challenges the teachers [4].

In the USA, in order to promote students’ computer programming skills, the Next Generation Science Standards [5] adopted CT as one of the core disciplines for teaching STEM (Science, Technology, Engineering, and Mathematics). STEM is fundamental to learning computer science. However, it is not easy to enhance students’ STEM ability, even for the electronic or engineer colleges, which are heavily connected to the computer science department. Although teachers dedicate themselves to enhancing students’ STEM ability, students still struggle to learn the content of computer programming due to the nature of the subject itself [4]. Students without a major in computer science struggle even further, of course: because they have not taken sufficient courses to cultivate their STEM ability, they struggle to learn the concepts of computer programming [6]. Moreover, they are learning about an unfamiliar field, so they do not have sufficient motivation to complete the course [7].

Hence, in order to enhance students’ motivation to facilitate their STEM ability, it is important to survey and understand students’ computer perceptions first and afterwards use effective teaching strategies to facilitate students’ learning. To reach the goal to understand students’ perceived competence, the study used Chung Shan Medical University as the research site. The university has paid attention to AI development, hence, it enacts C++ computer programming language course for the freshmen. However, this is a big challenge for students and teachers, because these students do not have sufficient courses related to STEM.

## 2. Literature Review

Artificial intelligence (AI) has gradually contributed to the creation of numerous products that people will use throughout their life. The level of AI will heavily impact a national’s competitiveness. Hence, many countries seek to cultivate scientific and technological capabilities in the field of AI. Computational thinking (CT) also offers great advantages for society. In 2016, the US White House administration’s plan of “Computer Science for All” emphasized CT, which is a requisite skill for students since it will create a digital economy for a thriving USA [8]. Naturally, successful AI is reliant upon one’s skill in computer programming; hence, it is essential that students be motivated to develop their CT in computer programming learning.

It is worth noting that CT is not synonymous with computer programming, although it is an important factor for students’ learning of computer programming [9]. Many instructors teach the concepts of CT, the practices of CT, and the perspectives of CT because computer programming plays an important role in the application of CT [10,11]. Some studies have proven that the use of visual programming tools to teach CT yields positive performance results. Bers et al. [12] designed six lessons involving the CT curriculum and evaluated students’ CT concepts via a six-point Likert scale after the final class. These lessons included engineering design, robotics, sequencing, sensors, and looping and branching. The students scored 4 or higher, achieving the target level. Berland and Wilensky [13] designed physical and virtual versions of complex system algorithms and CT in the curriculum. Their results showed good performance in computer programming and system thinking.

Kalelioglu et al. [14] pointed out that the accepted definition of CT is still in the process of being explored. However, many scholars and educators have agreed that CT could involve the concepts of loops, events, sequences, parallelism, etc. [15,16,17]. Brennan and Resnick [18] defined CT in terms of three dimensions: computational concept, computational practice, and computational perspective. “Computational concept” relates to the fundamental concepts about computer science, for instance, loops, conditionals, and so forth. “Computational practice” regards abstraction, debugging, and iteration. “Computational perspective” pertains to expressing, connecting, and questioning in programming. Thus, to improve students’ CT, computer programming should be connected to math and science learning [19,20].

Despite the benefits and importance of CT, many students are experiencing difficulties in learning computer programming, which, of course, may lead to low learning performance and satisfaction levels [21,22,23,24]. They may also struggle with syntactic errors. Sometimes, the errors are from complex logical structures. For example, students encounter abstraction and complexity when trying to understand the concepts of variables, loops, and functions. Berssanette and de Francisco [25] obtained some categories for the difficulties which may result from several areas, including the errors of reading and interpretation, the complexity of logical reasoning, deficient problem-solving skills, and so on. The problems regarding students’ learning difficulties should be a focus of attention. The failure rate of introductory programming subjects is about 30% [26]. It means that the difficulties will result in students’ learning bottlenecks in computing and informatics subjects. Moreover, these difficulties will hinder students from continuing learning in relative computer courses. Ozoran, Cagiltay, and Topalli [27] pointed out that these difficulties may hinder students’ learning as they chase computer programming success. The term “chase” refers to students’ attitudes and self-efficacy, which will be affected by these difficulties [28,29].

Students’ learning attitudes will also affect their emotional reactions and behaviors, as well as their engagement. The term “learning attitude” pertains to the learning of a task or a value. It can be defined by the cognitive process and active and emotional engagement of students in a particular learning process [30,31]. Some researchers [32,33] determined that three interlocking factors affect students’ engagement and academic performance. The three factors are behavior, emotion, and cognition.

Hence, to gain competence in computer programming, students’ attitudes and their self-efficacy beliefs are essential [28,34,35]. Baser [28] and Korkmaz and Altun [35] showed that there is a significant difference between students’ learning attitudes toward computer programming and learning goals. If students’ perceptions are negative regarding their self-efficacy in learning computer programming, they may not be able to pass these courses [28,34].

The concept of empowerment involves one’s ability to manage their lives and their concerns [36]. Some studies have determined that there is only one component in empowerment. Sprague and Hayes’s research [37] showed that component is self-determination. Breton [38] thought it was competence, and Larson, Walker, and Pearce [39] believed it was self-confidence. Other studies have stated that empowerment has multiple components. Thomas and Velthouse [40], Hur [41], and Spreitzer [42] found four components involved in empowerment: impact, competence, choice, and meaningfulness. To examine students’ empowerment, three of these components play a significant role: meaningfulness, impact, and competence [43]. The other component, choice, has not been found to apply to students’ learning [44,45] because, according to Frymier et al. [43], students are typically not given a choice in the curriculum design.

Students can show their creativity through CT [18]. Brennan and Resnick [18] put forward a method for examining the perceived creativity of students. The researchers did not focus on self-determination/choice. However, they believed that PE had occurred, and that students feel empowered based on four factors: (1) the computer programming is meaningful, (2) they are impacted by the need to complete the tasks, (3) they value their creativity to complete their tasks, and (4) self-efficacy helps them to complete their tasks. These four factors will be further detailed below.

“Meaningfulness” means that people can perceive the task’s value according to their own ideals or goals [46,47]. Hence, if students perceive learning computer programming to be an important task for their future, then learning computer programming becomes meaningful. As a result, these students will feel more empowered than others [43]. That is, when students view learning computer programming as meaningful, compared with other students, they are more likely to start learning it, devote themselves to completing it, and overcome obstacles to achieve success [42]. Thus, students are not empowered only because something is “meaningful,” but because it is for them. Even though sometimes, something can be meaningful, students perceive that other profiles can do it better than them, and therefore, they do not feel empowered. Thus, in order to enable students to feel empowered, some research has showed that students can learn as designers or creators, such as designing computer games [48] or getting involved in project-based learning [49].

Frymier et al. [43] defined “impact” as occurring when the “accomplishment of a task is perceived to make a difference in the scheme of things” (pp. 183–184). If someone feels that completing a task will have a great impact, he/she will have more motivation to accomplish it. In learning computer programming, students should feel that they will transform their roles from being a user of computer science to a generator of computer technology [50]. Brennan and Resnick [18] thought this transformative situation could promote student empowerment by impacting their career development.

Brennan and Resnick [18] showed that students believe in a connection between creativity and self-efficacy because they believe they can produce new ideas and solutions. Hence, when someone faces an obstacle, greater creative self-efficacy will bring about different ideas and methods, leading to success [51]. Self-efficacy is a type of belief. It means that someone believes that he/she has the technical skills and competencies necessary to complete a task, in this case, a computer programming task [41]. Deschryver and Yadav [52] and Voogt et al. [53] considered that CT education may stimulate students’ creativity, thereby empowering students to create products through the development of computer programming. Burke [54] indicated that a good computer programming learning climate can promote learners’ creative self-efficacy. Hence, when students believe themselves to be creatively successful with computer programming tasks, they may feel empowered [43], and thus can accomplish a task with less difficulty. The four empowerment components—meaningfulness, impact, creative self-efficacy, and programming self-efficacy—can increase the possibility of accomplishing a task. Students’ concept of empowerment will promote their willingness to learn computer programming.

## 3. Research Motivation

Chung Shan Medical University has enrolled first-year students in computer courses in order to prompt students’ skill and knowledge of computer programming. Each freshman must learn the C++ computer programming language by taking 2 credits in one semester (18 weeks). However, these students have not taken enough related courses to learn the unfamiliar field of computer programming because they are majoring in the medical field. Hence, in order to understand the learning effect of students who think with intention, their learning attitude, CT perspectives, and PE were explored after they took a C++ computer programming language course.

## 4. Research Model and Hypotheses Development

“Attitude” is defined as “how much we like (and/or dislike) something” by Maio, Haddock, and Verplanken ([55], p. xv). Therefore, people’s attitudes vary based on several factors. Students’ CPLA may affect their CT perspective and PE. For students’ computer programming learning, strong cognitive skills may directly relate to CPLA and demonstrate their self-efficacy beliefs [28,35]. Hence, if one person believes in his/her ability to complete a task, this person will address the problem with a positive attitude and willingness [56,57]. However, if an individual has a negative attitude/perception, this person could fail to learn computer programming [34]. For these reasons, this paper constructed the following hypotheses for CPLA and CT perspectives:

**H1:**  *Willingness affects computational thinking perspectives.*

**  H1a:**  *Willingness affects the ability to express.*

**  H1b:**  *Willingness affects the ability to connect.*

**  H1c:**  *Willingness affects the ability to question.*

**H2:**  *Negativity affects computational thinking perspectives.*

**  H2a:**  *Negativity affects the ability to express.*

**  H2b:**  *Negativity affects the ability to connect.*

**  H2c:**  *Negativity affects the ability to question.*

**H3:**  *Necessity affects computational thinking perspectives.*

**  H3a:**  *Necessity affects the ability to express.*

**  H3b:**  *Necessity affects the ability to connect.*

**  H3c:**  *Necessity affects the ability to question.*

In addition, students’ positive interest may affect their PE. Hidi [58] noted that interest is a type of psychological state. Students will demonstrate concentration and positive cognition if they are interested in an activity. Hence, if students are interested in programming, they will have a predisposition for programming activities. Furthermore, their interest is in accordance with their learning attitude. Furthermore, their interest is in accordance with their learning attitude, which is associated with a sense of enjoyment [59]. With a positive learning attitude, students may quickly develop PE. Weber, Martin, and Cayanus [60] pointed out that attaining PE involves learning more about programming’s meaningfulness, impact, creative self-efficacy, and programming self-efficacy. Hence, the following hypotheses are established:

**H4:**  *Willingness has an effect on PE.*

**  H4a:**  *Willingness has an effect on meaningfulness.*

**  H4b:**  *Willingness has an effect on impact.*

**  H4c:**  *Willingness has an effect on creative self-efficacy.*

**  H4d:**  *Willingness has an effect on programming self-efficacy.*

**H5:**  *Negativity has an effect on PE.*

**  H5a:**  *Negativity has an effect on meaningfulness.*

**  H5b:**  *Negativity has an effect on impact.*

**  H5c:**  *Negativity has an effect on creative self-efficacy.*

**  H5d:**  *Negativity has an effect on programming self-efficacy.*

**H6:**  *Necessity has an effect on PE.*

**  H6a:**  *Necessity has an effect on meaningfulness.*

**  H6b:**  *Necessity has an effect on impact.*

**  H6c:**  *Necessity has an effect on creative self-efficacy.*

**  H6d:**  *Necessity has an effect on programming self-efficacy.*

## 5. Research Design

This study investigated medical school students’ CPLA, CT perspectives, and PE in learning the computer programming of C++ language. A total of 495 samples were obtained. The students participating in the study were derived from random sampling. Furthermore, this study was approved by the guidelines of the Institutional Review Board of Chung Shang Medical University Hospital (No. CS1-20034); moreover, before they filled in the scales, they had been informed of the research purposes. Each student completed three scales: the Computer Programming Learning Attitude scale (CPLAS), the CT Perspectives scale (CTPS), and the Programming Empowerment scale (PES). The statistical software, Amos 24 [61], was used for the confirmatory factor analysis (CFA) and Structural equation modeling (SEM).

### 5.1. Instruments

**Figure A1:**
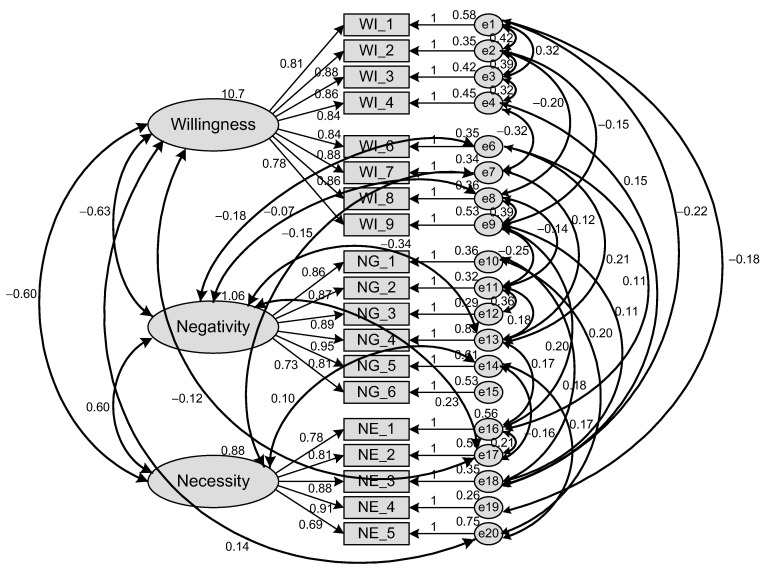
Final confirmatory factor analysis of CPLAS.

The five-point Likert-type CPLAS scale was developed by Korkmaz and Altun [62]. It is composed of three factors: willingness (nine items), negativity (six items), and necessity (five items) in CPLAS. The CPLAS items for each factor are shown in Figure A1 of Appendix A. The reliability of CPLAS was between 0.749 and 0.824 for the Cronbach alpha reliability coefficient. Yukselturk and Altiok [63] also verified CPLAS in their study, and the reliability coefficient of the Cronbach alpha was between 0.900 and 0.928.

**Figure A2:**
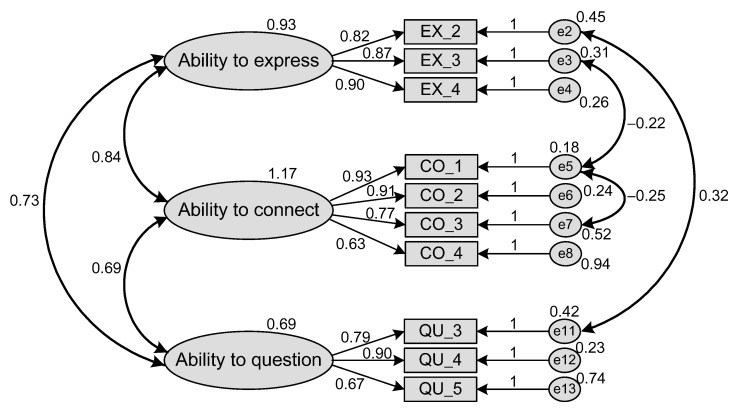
Final confirmatory factor analysis of CTPS.

In addition, Kong and Wang’s [64] CTPS, with a five-point Likert scale, was used in this study. In addition, Kong and Wang’s [64] five-point Likert CTPS, based on Brennan and Resnick’ framework [18], was used in this study. There are three factors included in the CTPS: the ability to express (four items), the ability to connect (four items), and the ability to question (five items). The overall Cronbach alpha was 0.95, and the three factors’ Cronbach alphas were 0.95, 0.80, and 0.88 for factor 1, factor 2, and factor 3. The items in CTPS are shown in Figure A2 of Appendix A.

**Figure A3:**
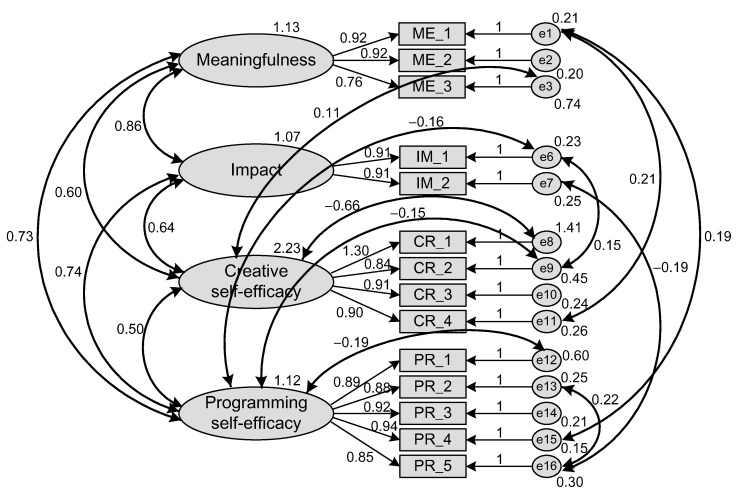
Final confirmatory factor analysis of PES.

PES, with a five-point Likert scale, was developed by Kong, Chiu, and Lai [65]. It is composed of four factors: meaningfulness (four items), impact (three items), creative self-efficacy (four items), and programming self-efficacy (five items). The reliability coefficient of the Cronbach alpha was 0.883 for meaningfulness, 0.878 for impact, 0.822 for creative self-efficacy, and 0.919 for programming self-efficacy. The items in PES are shown in Figure A3 of Appendix A.

### 5.2. Confirmatory Factor Analysis (CFA)

First, the fits of the three scales—CPLAS, CTPS, and PES—were tested separately. Table 1 displays the goodness-of- fit indices for the measurement model [66,67,68]. For CPLAS, because the factor loading for item WI_5 was 0.149 (less than 0.5), this item was deleted. Furthermore, the study increases the indices to reach the acceptable level of fit of the final CFA of the CPLAS, which is shown as Appendix A (Figure A1). For CTPS, because the factor loadings for items EX_1, QU_1, and QU_2 were 0.227, 0.195, and 0.312 (less than 0.5), respectively, they were deleted. Moreover, the study increases the indices to reach the acceptable level of fit of the final CFA of the CTPS, which is shown as Appendix A (Figure A2). For PES, because the factor loadings for items ME_4 and IM_1 were 0.225 and 0.244 (less than 0.5), respectively, they were deleted. Furthermore, the study increases the indices to reach the acceptable level of fit of the final CFA of the PES, which is shown as Appendix A (Figure A3). All of the RMSEs for the three scales were below 0.05; hence, these scales show a very good fit. Other indices also prove that these three scales have an acceptable level of fit. In short, the results of the indices concluded that these scales show an excellent fit to our data.

Furthermore, in order to evaluate the internal reliability, convergent validity, and discriminant validity in CPLAS, CTPS, and PES, Table 2 shows the items’ factor loading, Cronbach alpha, and composite reliability for CPLAS, CTPS, and PES. The internal reliability, Cronbach alpha, was between 0.832 and 0.953, which is greater than 0.7, the criterion for data with acceptable internal consistency [69,70]. Hence, the data have acceptable internal consistency. Convergent validity was checked by average variance extracted (AVE), composite reliability, and item loadings. As shown in Table 1, the composite reliability was between 0.832 and 0.954, which reveals a strong internal reliability for these obtained data [71]. The individual item loading was between 0.630 and 0.999 (simultaneously, the factor loadings have statistical significance, *p* < 0.001), whereas the values of AVE were between 0.790 and 0.916 (see Table 3, Table 4 and Table 5), exceeding the recommended level of 0.5 [67,72]. Hence, the convergent validity for these scales is fulfilled. Furthermore, discriminant validity can be evaluated by comparing the factors’ √AVEs with the corresponding factors’ correlation coefficient. If the two factors’ √AVEs are higher than the two factors’ correlation coefficients, discriminant validity has been proven [72]. In Table 3, Table 4 and Table 5, all √AVEs are greater than their corresponding two factors’ correlation coefficients; hence, the discriminant validity is proven in these three scales.

## 6. Results

**Figure 1:**
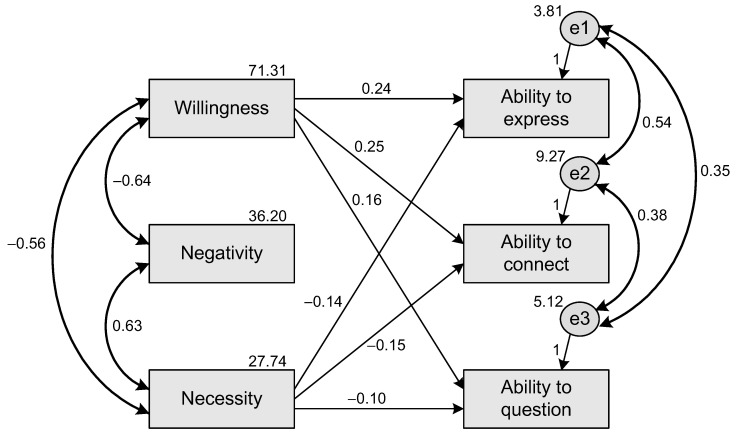
The SEM of CPLA to CT perspectives.

**Figure 2:**
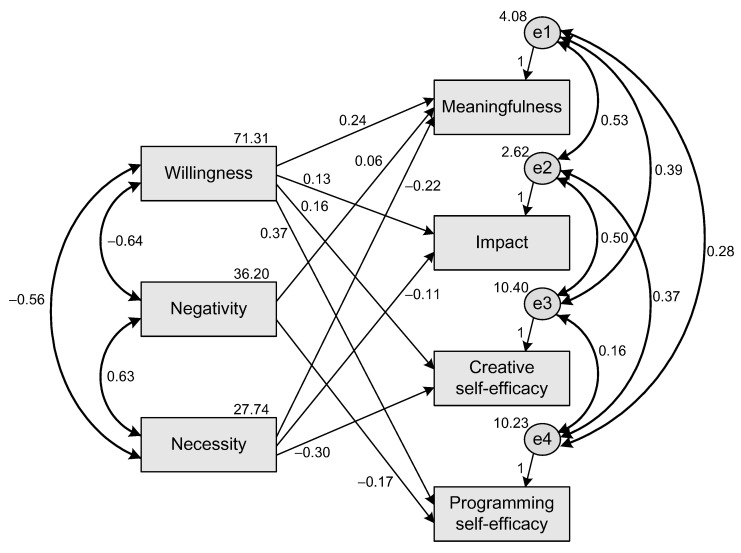
The SEM of CPLA to PE.

To test the above hypotheses, SEMs were constructed, and nonsignificant paths were removed. The SEMs’ goodness of fit were also evaluated; the criteria of indices are shown in Table 1. The results of the SEMs are shown in Figure 1 and Figure 2.

### 6.1. The SEM of CPLA to CT Perspectives

Figure 1 shows the SEM of CPLA to CT perspectives. The obtained values of the indices are as follows: X^2^/df = 1.878 (*p* = 0.131 > 0.05), RMSEA = 0.047, GFI = 0.995, AGFI = 0.967, NFI = 0.996, RFI = 0.980, IFI = 0.998, TLI = 0.991, and CFI = 0.998. A comparison of the criteria of the indices (acceptable level of fit) in Table 1 shows that the hypothesized model has goodness-of-fit data. Indeed, the hypothesized model shows that there is a significant positive effect in the paths of willingness regarding the factors of CT perspectives (H1a, H1b, and H1c). The H1a and H1b results are almost the same: willingness has a positive effect on students’ ability to express (Beta = 0.24, *p* < 0.000) and to connect (Beta = 0.25, *p* < 0.000). However, in H1c, willingness has a less positive effect on the ability to question (Beta = 0.16, *p* < 0.000) compared with the other results. For H2a, H2b, and H2c, there is no significant (all *p*s > 0.05) support for the paths between negativity and the factors of CT perspectives. For H3a, H3b, and H3c, there is significant (all *p*s < 0.000) support for the paths between necessity and the factors of CT perspectives. They are almost the same regarding the negative effect between the path of necessity and the ability to express (H3a, Beta = −0.14, *p* < 0.000), and the path of necessity and the ability to connect (H3b, Beta = −0.15, *p* < 0.000). The path of necessity and the ability to question (H3c, Beta = −0.10, *p* < 0.000) has a smaller negative effect than those of the other two paths (H3a, H3b).

### 6.2. The SEM of CPLA to PE

Figure 2 shows the SEM of computer programming learning attitude to PE. The obtained values of the indices are as follows: X^2^/df = 1.571 (*p* = 0.179 > 0.05), RMSEA = 0.038, GFI = 0.995, AGFI = 0.968, NFI = 0.997, RFI = 0.982, IFI = 0.999, TLI = 0.993, and CFI = 0.999. A comparison of the acceptable levels of fit in Table 1 shows that the hypothesized model has goodness-of-fit data. This model shows that there is significant support for the positive effects in the paths of willingness to the factors of PE (H4a, H4b, H4c, and H4d). Students demonstrated the strongest positive effect from willingness to programming self-efficacy (H4d, Beta = 0.37, *p* < 0.000) compared with the positive effects of other paths (H4a, H4b, H4c). The second strong positive effect is from willingness to meaningfulness (H4a, Beta = 0.24, *p* < 0.000). The positive effects for the paths from willingness to impact and to creative self-efficacy are 0.13 (H4b, *p* < 0.000) and 0.16 (H4c, *p* < 0.000). For the paths from negativity to the factors of PE, two paths—from negativity to meaningfulness and from negativity to programming self-efficacy—are significant (H5a, Beta = 0.065, *p* = 0.002 < 0.05; H5d, Beta = −0.166, *p* < 0.000). However, the paths from negativity to impact (H5b) and from negativity to creative self-efficacy (H5c) are not significant (*p*s > 0.05). For the paths from necessity to the factors of PE, H6c, the path from necessity to creative self-efficacy (Beta = −0.301, *p* < 0.000) has the strongest negative effects compared with the others (H6a, H6b). The negative effect from necessity to meaningfulness (H6a) is −0.221 (*p* < 0.000) and from necessity to impact (H6b) is −0.110 (*p* < 0.000). For the path from necessity to programming self-efficacy, the effect is nonsignificant (*p* > 0.05).

## 7. Discussion

In this paper, the first focus is on the development of three scales—CPLAS, CTPS, and PES—and their validation. The results of the CFA prove that the three scales have goodness of fit. Overall, three factors were derived from the CPLAS: willingness (eight items), negativity (six items), and necessity (five items). In the CTPS, there are three factors: the ability to express (three items), ability to connect (four items), and ability to question (three items). For PES, there are four factors in the scale: meaningfulness (three items), impact (two items), creative self-efficacy (four items), and programming self-efficacy (five items). The factor loadings and reliability prove that the three scales’ factors have enough convergent validity. Furthermore, the AVE for all of the scales’ factors confirm their discriminant validity.

After analyzing the structure from students’ CPLA to their CT perspectives, two factors of CPLAS, willingness and necessity, have significant effects (*p* < 0.000). However, the negativity factor of CPLAS does not have significant effects (*p* > 0.05) toward the CT perspectives, meaning that negative thinking cannot improve students’ CT perspectives, including their ability to express, connect, and question. However, positive thinking and willingness will improve students’ CT perspectives. Kim and Kim [73] found that reducing mental and cognitive loading in learning programming education by reducing students’ negative attitudes will lead to a significant increase in students’ computation and cognitive CT perspective. Further, regarding the CT perspectives’ factors, including the ability to express, connect, and question, a willing learning attitude is stronger than a learning attitude geared toward necessity. Willingness shows a positive effect on CT perspectives. However, necessity shows a negative effect on CT perspectives. The reason may be because the students of the Chung Shan Medical University had not taken enough related courses to learn the unfamiliar field of computer programming. Moreover, the course is mandatory (compulsory credits) for each freshman. Hence, students who feel willingness show a positive effect on their CT perspective. If they recognize the necessity but feel forced to attend this course, a negative effect results regarding their CT perspective. Pollock et al. [74] emphasized that students sometimes cannot understand and appreciate why they must learn computer programming. Thus, teachers must emphasize the importance of disciplinary-specific courses in order to integrate and increase students’ learning motivation in order to cultivate positive learning attitudes. In addition, with respect to the CT perspective, learning attitudes related to willingness and necessity have stronger effects on the ability to express and connect than the ability to question. This phenomenon shows that when learning computer programming, freshmen are more likely to adopt the express perspective and connect perspective and less likely to adopt the question perspective. That is, from the definition of the ability to express, ability to connect, and ability to question [18], students think about (1) how to apply their learning to problem-solving (ability to express), and (2) how to cooperate with their classmates in collaborative programming activities (ability to connect), based on their CPLA. They devote little attention to asking questions (ability to question) because they are only learning the C language for 2 credits for one semester. Kong et al. [65] noted that if students are lacking PE, they may not feel able to devise questions in computer programming. However, pertaining to the ability to express and to connect, students’ cognition would benefit from the suggestions of Ke [75] and Roth and Brooks-Gumn [76]. They suggested that teachers encourage and provide feedback to improve students’ positive attitude, thereby helping students to enhance their skills in programming learning by sharing their knowledge and discussing matters with their classmates.

In addition, analyzing the structure from students’ CPLA to their PE, a willing learning attitude has a significant positive effect (*p*s < 0.000) on all factors of PE. A negative learning attitude has a significant positive effect (*p* = 0.002 < 0.05) on meaningfulness and a significant negative effect (*p* < 0.000) on programming self-efficacy. A necessity-based learning attitude has a significant positive effect (*p*s < 0.000) on meaningfulness, impact, and creative self-efficacy. Hence, willingness can cultivate students’ positive learning attitude toward engaging in programming learning activities [77]. For the meaningfulness PE, a stronger positive attitude will affect their work (meaningfulness) due to a strong emotional attachment ([78], p. 108). If students have a willing and positive learning attitude, meaningfulness will be affected positively. However, if students feel that the course is an obligation (compulsory credits), they may feel a negative emotion. Necessity will produce a negative learning attitude effect on meaningfulness. In addition, the definition of “impact” in this paper is to attempt a task. In addition, the impact involves that the completion of a task may bring a difference to how the things are organized and related to each other [43]. Hence, for the impact PE, a positive and willing learning attitude has a positive effect, but necessity has a negative effect. For creative self-efficacy PE, from the view of social cognitive theory, Tantawy et al. [79] found that attitudes and creative process engagement provide positive mediation for creative self-efficacy, based on students who took seven entrepreneurship courses. Michael, Hou, and Fan [80] determined that creative self-efficacy is an optimal moderator between individual psychological traits and innovation behavior. That is, enhancing creative self-efficacy will improve innovation behavior in work. Hence, improving positive individual psychological traits, that is, individual work attitude, is important. In this study, a willing learning attitude has a positive effect on impact, thereby enhancing students’ willingness to learn computation programming, which could encourage students’ innovation behaviors. For programming self-efficacy, Korkmaz [81] investigated students’ attitudes in connection with learning computer programming, self-efficacy, and academic achievement. Their result was interesting: if students thought learning C++ programming was irrelevant for their task, they showed a weak self-efficacy belief. This situation is similar to the students of the Chung Shan Medical University. If they feel their future work is not highly related to learning computer programming, a negative learning attitude may occur. Hence, a negative learning attitude has a negative effect on programming self-efficacy. On the contrary, Özyurt [82] studied a computer programming course in distance education for students, considering their learning attitude regarding programming self-efficacy and programming. He found that there was a positive, statistically significant connection between students’ learning attitude and programming self-efficacy. Thus, a willing learning attitude will positively prompt students in programming self-efficacy.

Based on the above discussion, students’ positive attitude will increase their CT perspectives and PE. Thus, for students of the Chung Shan Medical University, methods to increase students’ positive CPLA are worth further discussion. Korkmaz [29] and Wang et al. [83] found computer programming to be complex for students’ learning. Thus, it is essential that students’ CPLA, CT perspective, and PE improve. In this study, regarding enhancing students’ learning effectively for the C++ programming language, some studies are worthy of note. Sengupta et al. [84] emphasized that integrating the teaching curriculum, including computing in math and sciences, in order to help students to learn logic coherently, since it will promote students’ learning development. In addition, some researchers studied how learning computer programming influenced students’ learning attitude and further affected their CT. Mason and Rich [85] found that a curriculum design that implements students’ coding ability results in students’ learning success with coding and increases their CPLA, fostering student growth. Repenning et al. [86] emphasized the pedagogical design in order to lead to students’ understanding in programming algorithmic constructs. Garneli and Chorianopoulos [87] supported the concept of learning strategies, which prompt students’ learning in computer programming. These studies can benefit the Chung Shan Medical University in promoting students’ learning of the C++ computer programming language through an adaptive curriculum, pedagogy, and learning strategies in order to promote students’ CPLA.

## 8. Conclusions

In this study, the authors revised CPLAS, CTPS, and PES by using CFA via SEMs to perceive participants’ learning attitudes, thinking perspectives, and perceived empowerment. Thus, the CFA’s results demonstrated that CPLAS, CTPS, and PES have good reliabilities, convergent validities, and discriminant validities. For the results of SEMs, the results show that a positive CPLA will have a positive effect on CT perspectives and PE. However, if students feel that they are forced to learn the C++ computer programming language, a negative CPLA will have a negative effect on CT perspectives and PE.

This study is limited because the samples are from a medical university, and the students only had 36 h (2 credits of one semester) of instruction for the C++ computer programming language in their first year. Hence, to compare these results to other colleges, such as business or engineering, readers must understand the research background. The improvement of students’ CPLA will be a key direction for further research. Lastly, for the Chung Shan Medical University and the subject of the C++ computer programming language, three different facets, i.e., teaching strategies, teaching curriculum design, and pedagogy design, could be explored in further studies. In addition, future study could take students’ choice into consideration to develop curriculum design, which may match students’ learning interests in different computer programming. The curriculum design may also use online courses to facilitate students’ adaptive learning.

## Figures and Tables

**Table 1:** Goodness-of-fit indices for CPLAS, CTPS, and PES.

Type of Measure	Acceptable Level of Fit	CPLAS	CTPS	PES
Chi-square/degree of freedom	≤3.5 to 0 (perfect fit) and (*p* > 0.01)	1.23, *p* = 0.048	1.109, *p* = 0.312	1.367, *p* = 0.030
Root-Mean Residual (RMR)	Less than 0.05	0.040	0.031	0.037
Root-Mean Square Error of Approximation (RMSEA)	Below 0.1 (a good fit): below 0.05 (a very good fit)	0.040	0.017	0.031
GFI	Greater or equal to 0.9	0.996	0.984	0.972
AGFI	Greater or equal to 0.9	0.941	0.969	0.951
Normed Fit Index (NFI)	Greater or equal to 0.9	0.980	0.989	0.985
Relative Fit Index (RFI)	Greater or equal to 0.9	0.970	0.982	0.977
Incremental Fit index (IFI)	Greater or equal to 0.9	0.996	0.999	0.996
Tucker Lewis Index (TLI)	Greater or equal to 0.9	0.994	0.998	0.994
Comparative Fit Index (CFI)	Greater or equal to 0.9	0.996	0.999	0.996

**Table 2:** The measurement model.

Factor	Items	Factor Loading	t	Composite Reliability	Cronbach’s Alpha
CPLAS: computer programming learning attitude scale					
Willingness	WI_1	0.806		0.952	0.954
	WI_2	0.875	27.072 **		
	WI_3	0.860	24.653 **		
	WI_4	0.839	19.573 **		
	WI_6	0.842	19.720 **		
	WI_7	0.883	21.054 **		
	WI_8	0.859	20.316 **		
	WI_9	0.777	17.702 **		
Negativity	NG_1	0.864		0.942	0.919
	NG_2	0.873	22.644 **		
	NG_3	0.885	23.305 **		
	NG_4	0.949	15.372 **		
	NG_5	0.808	19.847 **		
	NG_6	0.732	17.084 **		
Necessity	NE_1	0.781		0.909	0.910
	NE_2	0.809	20.122 **		
	NE_3	0.880	19.517 **		
	NE_4	0.911	20.716 **		
	NE_5	0.692	14.532 **		
CTPS: CT perspectives scale					
Ability to express	EX_2	0.822		0.900	0.900
	EX_3	0.872	21.008 **		
	EX_4	0.904	21.850 **		
Ability to connect	CO_1	0.931		0.889	0.875
	CO_2	0.910	29.021 **		
	CO_3	0.774	18.750 **		
	CO_4	0.630	14.600 **		
Ability to question	QU_3	0.787		0.832	0.821
	QU_4	0.896	17.685 **		
	QU_5	0.674	13.501 **		
PES: programming empowerment scale					
Meaningfulness	ME_1	0.919		0.902	0.894
	ME_2	0.920	29.575 **		
	ME_3	0.760	19.783 **		
Impact	IM_2	0.908		0.903	0.901
	IM_3	0.906	26.952 **		
Creative self-efficacy	CR_1	0.999		0.954	0.891
	CR_2	0.843	13.697 **		
	CR_3	0.910	14.429 **		
	CR_4	0.905	14.359 **		
Programming self-efficacy	PR_1	0.890		0.953	0.941
	PR_2	0.881	17.436 **		
	PR_3	0.918	18.221 **		
	PR_4	0.939	18.673 **		
	PR_5	0.853	16.883 **		

** *p* < 0.001.

**Table 3:** Average variance extracted, square root of AVE, and matrix of correlations between factors for CPLAS.

Factor	AVE	1	2	3
1. Willingness	0.711	**0.843**		
2. Negativity	0.730	−0.640 **	**0.854**	
3. Necessity	0.670	−0.560 **	0.632 **	**0.819**

The values shown in bold are the square root of AVE; ** *p* < 0.001, total AVE = 0.706.

**Table 4:** Average variance extracted, square root of AVE, and matrix of correlations between factors for CTPS.

Factor	AVE	1	2	3
1. Ability to express	0.751	**0.867**		
2. Ability to connect	0.673	0.769 **	**0.820**	
3. Ability to question	0.625	0.648 **	0.623 **	**0.790**

The values shown in bold are the square root of AVE; ** *p* < 0.001, total AVE = 0.682.

**Table 5:** Average variance extracted, square root of AVE, and matrix of correlations between factors for PES.

Factor	AVE	1	2	3	4
1. Meaningfulness	0.756	**0.869**			
2. Impact	0.823	0.775 **	**0.907**		
3. Creative self-efficacy	0.839	0.673 **	0.704 **	**0.916**	
4. Programming self-efficacy	0.804	0.673 **	0.667 **	0.506 **	**0.897**

The values shown in bold are the square root of AVE; ** *p* < 0.001, total AVE = 0.807.
